# Bridging Aging and Disability Research: Where to Go From Here?

**DOI:** 10.1093/geroni/igaf122.975

**Published:** 2025-12-31

**Authors:** Michelle Putnam, Susan Stark

## Abstract

This session discusses future directions for bridging aging and disability research, building from their work published in a special issue of The Gerontologist. The first presenter will provide a historical overview of efforts to bridge aging and intellectual disability, discussing trends in population aging, research and policy initiatives that have forwarded bridging over time, and examples of evidence-based knowledge and practice interventions that demonstrate realized outcomes of bridged aging and disability research such as future planning and health promotion and areas where more bridged research is needed including areas of family support. The second presenter will discuss the TechSage Technology Intervention Model, which designs for the intersection of aging and disability, emphasizing interdisciplinary problem solving. Looking forward to new technologies, opportunities and challenges to utilizing this model and implications and recommendations for bridging aging and disability research will be identified. The third presenter will discuss the evolution of aging and disability research over time, conceptual and methodological challenges to engaging in research that bridges aging and disability domains, and opportunities for reconciling these to support future bridging work. The session’s discussant will reflect upon where bridging work goes from here by highlighting gaps that need to be addressed, examples of success, and considering how researchers can more actively engage in bridging research work. Lifelong Disabilities Interest Group Sponsored Symposium

